# COVID-19 Vaccines in Children with Cow’s Milk and Food Allergies

**DOI:** 10.3390/nu13082637

**Published:** 2021-07-30

**Authors:** Lucia Liotti, Annamaria Bianchi, Paolo Bottau, Silvia Caimmi, Giuseppe Crisafulli, Fabrizio Franceschini, Francesca Mori, Claudia Paglialunga, Francesca Saretta, Carlo Caffarelli

**Affiliations:** 1 Department of Pediatrics, AOU Ospedali Riuniti Ancona, Presidio Ospedaliero di Alta Specializzazione “G. Salesi”, 60100 Ancona, Italy; lucia.liotti@ospedaliriuniti.marche.it (L.L.); allped@libero.it (F.F.); 2 UOC Pediatria, Azienda Ospedaliera Ospedale San Camillo Forlanini, 00152 Roma, Italy; annamaria.bianchi9@yahoo.it; 3 Pediatric and Neonatology Unit, Imola Hospital, 40026 Imola, Italy; p.bottau@gmail.com; 4 Dipartimento di Pediatria, Fondazione IRCCS Policlinico San Matteo, Università di Pavia, 27100 Pavia, Italy; sissi_del_78@hotmail.com; 5 UOC Allergologia, Dipartimento Materno-Infantile, Università di Messina, 98124 Messina, Italy; crisafullig@unime.it; 6 Allergy Unit, Department of Pediatrics, Meyer Children’s University Hospital, 50134 Florence, Italy; francesca.mori@meyer.it; 7 Pediatric Department, Azienda Ospedaliera-Universitaria “Consorziale-Policlinico”, Ospedale Pediatrico Giovanni XXIII, 70126 Bari, Italy; clapag07@gmail.com; 8 SC Pediatria, Ospedale Latisana-Palmanova, Dipartimento Materno-Infantile Azienda Sanitaria Universitaria Friuli Centrale, 33100 Udine, Italy; francescasaretta@gmail.com; 9 Clinica Pediatrica, Dipartimento di Medicina e Chirurgia, Università di Parma, 43126 Parma, Italy

**Keywords:** COVID-19, cow’s milk allergy, food allergy, vaccine, anaphylaxis, SARS-CoV-2

## Abstract

The COVID-19 pandemic is the most challenging global health crisis of our times. Vaccination against COVID-19 plays a key role to control the current pandemic situation. The risk of allergic reactions to new COVID-19 vaccines is low. However, there is a debate on the safety in allergic patients following post marketing findings by different agencies. Our aim is to understand from current experiences whether children with cow’s milk or food allergy are at higher risk than a general population for allergic reactions to COVID-19 vaccines. Current data indicate that patients with a history of allergy to cow’s milk or other foods, even if severe, should receive COVID-19 vaccine in a setting with availability of treatments for anaphylactic reactions and under medical supervision. Recipients should be discharged after a protracted observation period of 30 min if no reaction developed.

## 1. Introduction

The severe acute respiratory syndrome coronavirus-2 (SARS-CoV-2) has infected millions of individuals all over the world and it is the causative agent of coronavirus disease 2019 (COVID-19). The COVID-19 pandemic is the most challenging global health crisis of our times, with devastating health, social and economic impacts, as well as unpredictable long-term consequences in people of all ages. Children represent approximately 5% of those infected at the beginning of the pandemics in spring 2020, but more than 20% in spring 2021. Data show that children are less severely affected than adults. They have generally milder illness with atypical clinical manifestations. About 0.1–1.9% of all children with COVID-19 are admitted to hospital [1,2,3,4], showing low risk of complications and mortality [5]. However, several centers in Europe and United States have identified severe manifestations that can be associated with this infection, such as the Multisystem Inflammatory Syndrome in Children (MIS-C) [6,7]. Although the absolute risk of severe disease in children is low, children with comorbidities (as obesity, chronic cardiac or respiratory disease) have an increased relative risk [8]. Moreover, infants might be seriously ill. A large European multicenter study evidenced that 48% of pediatric patients infected with COVID-19 admitted to intensive care unit were under 2 years of age [9]. Currently, no drug effectively acts against SARS-CoV-2 [10]. In the global attempt to control the pandemic, COVID-19 vaccines play a critical role. Widespread vaccination against COVID-19 and its emerging variants with highly effective vaccines is an essential intervention to control the current pandemic situation. COVID-19 vaccines are not yet approved for use in young children, and their registration will be crucial to prevent the spread of the disease and to improve management of children with chronic diseases such as allergic diseases [11]. Cow’s milk allergy is the most common food allergy in children. There have been some reports that cow’s milk allergy or food allergy may hamper COVID-19 vaccination as it happens for other vaccines for infectious diseases. This may be a problem especially in children since cow’s milk and egg tolerance often develops before adulthood [12]. Our aim is to understand from current experiences whether the administration of COVID-19 vaccine in children with cow’s milk or food allergy can be risky and whether it may be necessary to take precautions.

## 2. COVID-19 Vaccines and Allergic Reactions

Allergic adverse reactions are possible after any type of vaccine for infectious diseases, but rarely occur. According to large population-based studies, anaphylaxis following vaccinations in an uncommon adverse reaction (less than 1 per million doses for most vaccines) [13,14,15,16], with no fatalities reported. Data used in the majority of the studies have been collected by passive surveillance systems to evaluate the risk of anaphylaxis after vaccination. Moreover, the exact rate of anaphylaxis for each vaccine is difficult to assess because vaccines are often simultaneously given. Data are constantly checked and updated [17]. Su et al. [18] recently analyzed Vaccine Adverse Event Reporting System (VAERS) database for anaphylaxis after vaccination in the US for a period of 27 years, up to 2016. In children and young adults (<19 age), MMR, Varicella and DTaP/Tdap vaccines were found to elicit most of the vaccine-induced anaphylactic reactions, while the influenza vaccine was the most frequently trigger in adults. The estimated rate of anaphylaxis during the 27 years period was found to be 0.2 per million doses of PCV23 and 0.6 per million doses of MMR. In a three year period (2009–2011), McNeil et al. [19], using Vaccine Safety Datalink, found that the rate of anaphylaxis was 1.31 per million vaccine doses administered. Adjuvants, stabilizers, animal proteins, active components, preservatives, antibiotics, and other compounds added during manufacturing can trigger allergic reactions to vaccines (Table 1) [20,21].

Two novel mRNA COVID-19 vaccines have been approved by various regulatory agencies [22]. Currently, Pfizer/BioNTech BNT162B2 is marketed for people over 12 years old, and Moderna mRNA-1273 for people over 16 years old. Two adenovirus-vector COVID-19 vaccines include Oxford-AstraZeneca, available for use in Europe and Johnson & Johnson’s Janssen that is available in the United States under Emergency Use Authorization. In phase 3 clinical trial, the new mRNA vaccines were generally well-tolerated [23]. Mild or moderate systemic adverse events generally occurred the day after the dose and lasted 1–2 days after vaccination [24]. In Pfizer-BioNTech trial, an anaphylactic reaction occurred [18]. However, large clinical trials, both for Pfizer-BioNTech and Moderna vaccines, excluded patients with “severe allergic reaction (e.g., anaphylaxis) to any component” of their vaccine [23,25]. The Pfizer/BioNTech trial for children aged 12–15 years has not registered allergic reactions [13].

Even if, the risk of allergic reactions to mRNA Covid-19 vaccine trials appeared to be very low, there is a debate on the safety in allergic patients following post marketing findings by national pharmacovigilance systems [25,26,27,28,29] (Table 2).

In US, the Centers for Disease Control and Prevention (CDC) and the U.S. Food and Drug Administration (FDA) conduct post-licensure safety monitoring of U.S. licensed vaccines by analyzing Vaccine Adverse Reporting System (VAERS) database [30]. The VAERS database of adverse events, including anaphylaxis, after vaccination passively receives information from vaccine recipients and/or healthcare providers. Similar spontaneous monitoring systems are also used by other countries. It is important to remember that data on adverse events of vaccination generated by voluntary reports must be accurately and prudently evaluated. They do not prove a cause-and-effect relationship for any adverse event, because of the intrinsic shortcomings of the system. In fact, the associations that they find, can turn out to be coincidental. Moreover, a diagnosis of clinical hypersensitivity reaction that relies only on self-reported symptoms is inaccurate. For example, symptoms like hot flashes, hypotension, passing out, and other symptoms that accompany anaphylaxis can be the result of a panic attack. “In vitro” and “in vivo” allergy tests are necessary to rule in or rule out that the occurrence of these symptoms was due to an episode of anaphylaxis.

On 9 December 2020, soon after starting the vaccination campaign, the British Medicines and Healthcare products Regulatory Agency (MHRA) reported two cases of anaphylaxis following the administration of Pfizer/BioNTech COVID-19 vaccine. A history of prior anaphylaxis was registered in both cases.

CDC preliminary analyzed VAERS data for the Pfizer-BioNTech COVID-19 vaccine from 14 to 23 December 2020 [26]. It reported 21 adverse events that met Brighton Collaboration criteria [31] for anaphylaxis after administration of 1,893,360 first doses of the Pfizer-BioNTech vaccine (11.1 cases per million doses). Symptoms occurred in 71% of cases within 15 min of vaccination and in 18 (86%) within 30 min [26]. No fatal events were reported. Among these 21 patients with anaphylaxis, 17 (81%) were managed in an emergency department and 4 were hospitalized (19%). A positive history of allergic reactions to drugs, insect stings or foods was found in 17 (81%) of 21 patients (19 females); a previous episode of anaphylaxis was reported in 7 patients (33%). Patients with previous allergic reaction had a history of food allergy in 3 cases (2 to eggs, 1 to cow’s milk, 1 to tropical fruit), those with previous anaphylaxis in 1 case (walnuts). As of 5 February 2021, an assessment of nationwide VAERS data reported 114 cases of anaphylaxis after administration of 19,582,865 doses of Pfizer-BioNTech with an anaphylaxis rate of 5.8 cases/million doses [32]. So, it seems that reported rate for anaphylaxis to Pfizer-BioNTech vaccine decreased with increased proportion of vaccinations.

The number of anaphylactic reactions to Moderna COVID-19 vaccine reported to VAERS [25] was 10 (all women) after administration of 4,041,396 doses of vaccine (2.5 cases per million doses) between 21 December 2020, and 10 January 2021. Among these 10 patients, 9 had previous allergic reactions. Only one out five patients with a history of anaphylaxis had anaphylaxis triggered by foods, but which foods were unspecified. As of 5 February 2021, the analysis of VAERS data showed 39 anaphylactic reactions to Moderna vaccine after administration of 17,133,644 doses with a rate of 2.3 cases/million doses [32].

From 27 December 2020, to 26 March 2021, 80 cases of anaphylaxis to COVID-19 vaccines were spontaneously submitted to AIFA, the Italian Medicine Agency [33]. Pfizer-BioNTech vaccine elicited 68 (85%) anaphylactic reactions (mostly in women following the first dose of vaccine), Moderna vaccine 2 (2.5%) reactions and Vaxzevria vaccine (Oxford-AstraZeneca) 10 (12.5%) reactions. The rate of anaphylaxis was 9.7 per million doses of Pfizer-BioNTech vaccine, 4.7 per million of Moderna vaccine and 6.1 per million of Vaxzevria vaccine. In 23 (29%) cases of anaphylaxis, patients had a prior history of non-specific allergic reaction to foods, inhalants, or insect stings; in 28 (35%) a prior drug allergy and in 5% a previous anaphylaxis [26]. A history of allergy to foods, insect stings or inhalants was registered for Pfizer-BioNTech, Moderna and Oxford-AstraZeneca vaccine in 29% (20/68) of cases, 50% (1/2), and 20% (2/10) respectively.

Regarding Oxford AstraZeneca vaccine, MHRA [34] registered 508 cases of anaphylactic reaction with one death, 87 anaphylactic shocks, 17 anaphylactoid reactions and 3 anaphylactoid shocks out of 23.3 million first dose as of 5 May 2021.

**Table 2:** Characteristics of reported cases of anaphylaxis following mRNA COVID-19 vaccine. * Combined data of food allergy, insect venoms, inhalants.

mRNA-COVID 19 Vaccine	Reference	Period Detected	Case of Anaphylaxis/ Doses Administered	Cases of Anaphylaxis/Million Doses	Sex	History of Allergic Reactions	History of Anaphylaxis	History of Food Allergy
Pfizer-BioNTech Vaccine	CDC COVID-19 Response Team; FDA [26]	14–23 December 2020	21/1,893,360	11.1	2 M 19 F	17/21 (81%)	7/21 (33%)	4/21 (19%) 1 tropical fruit, 1 eggs and milk, 1 eggs, 1 walnuts (with anaphylaxis)
	Desai 2021 [32]	14 December 2020–5 February 2021	114/19,582,865	5.8		49/114 (43%)	8/114 (7%)	
	Italian Medicine Agency 2021 [33]	27 December 2020–26 March 2021	68/6,994,739	9.7	6 M 62 F		4/68 (5,8%)	20/68 (29%) * unspecified food
	Blumenthal 2021 [35]	16 December 2020–12 February 2021	7/25,929	27 per 10,000 doses	1 M 6 F	3/7 (42.8%)	1/7 (14%)	
Moderna COVID 19 Vaccine	CDC COVID-19 Response Team; FDA [26]	21 December 2020–10 January 2021	10/4,041,396	2.5	10 F	9/10 (90%)	5/10 (50%)	1/10 (10%) unspecified food
	Desai 2021 [32]	14 December 2020–5 February 2021	39/17,133,644	2.3		21/39 (54%)	3/39 (7.7%)	
	Italian Medicine Agency 2021 [33]	27 December 2020–26 March 2021	2/427,731	4.7	0 M 2 F		0/2	1/68 (50%) * unspecified food
	Blumenthal 2021 [35]	16 December 2020–12 February 2021	9/38,971	23.1 per 10,000 doses	0 M 9 F	7/9 (77.7%)	4/9 (44.4%)	

At variance from above data resulting from passive voluntary surveillance systems, Blumenthal et al. [35] prospectively investigated for acute allergic reaction a cohort of 64,900 health care employees at Mass General Brigham (MGB) who received their first dose of a mRNA COVID-19 vaccine. Pfizer-BioNTech vaccine was given in 40% of cases and Moderna vaccine in 60% of instances. Anaphylaxis was confirmed in 16 (0.025%) recipients, 9 cases after Moderna vaccine and 7 cases after Pfizer-BioNTech vaccine. The incidence of anaphylaxis was estimated to be 27 cases per 100,000 doses of Pfizer-BioNTech vaccine and 23.1 cases per 100,000 doses of Moderna vaccine. All subjects were females, with the exception of one patient. In 10 (63%) subjects a prior allergy history was reported, and in 5 (31%) a prior anaphylaxis. A patient who reacted to Pfizer-BioNTech vaccine was allergic to tree nuts and shellfish. Three patients who reacted to Moderna vaccine were allergic to tree nuts, two to peanuts and one to shellfish. None of the patients required endotracheal intubation. Several explanations can be offered for higher proportion of anaphylactic reactions reported by Blumenthal et al. [35] compared to data from pharmacovigilance systems. Only a low proportion of adverse events following vaccinations following vaccinations are self-reported to health care professionals or to the surveillance system. Therefore, they may be underestimated. When people without medical education, voluntary submit adverse events to the surveillance system, scientific terms like “anaphylaxis” are not always used.

Overall, data from different sources seem to indicate that anaphylaxis to COVID-19 vaccines is more frequent than to vaccines for other infectious diseases.

The possible mechanisms underlying allergic reaction on first exposure to the mRNA COVID-19 vaccines are unclear [36]. In patients that have been previously sensitized to a vaccine component like PEG, release of mast cell mediators can be triggered by IgE antibodies to PEG. Mast cells can be also degranulated by preexisting anti-PEG IgG antibodies or by anaphylatoxins C3a and C5a generated during complement activation by anti-PEG IgM (or IgG) antibodies. Alternatively, anaphylaxis can be the result of a pseudoallergic response that does not need a prior exposure. In these reactions, PEGylated lipid nanoparticles encapsulating mRNA can activate mast cells or basophils directly or by complement activation with production of anaphylatoxins C3a and C5a [36]. It would be interesting to speculate on mechanisms associated with the development of immediate reactions to mRNACOVID-19 vaccines in patients with food allergy. It may be hypothesized that subjects with food allergy might be predisposed to develop pseudoallergic reactions. Studies of candidate genes associated with food allergy may be useful to comment further.

## 3. Vaccination in Patients with Food Allergy

Three important questions should be answered on the safety of COVID-19 vaccines in food allergic patients. Unfortunately, the answers to these important questions have not yet been addressed scientifically. Therefore, most of the recommendations are speculative. The first is whether the allergic reaction to COVID-19 vaccines can be related to food allergens contained in the vaccines. Excipients in the vaccine such as preservatives, antibiotics and adjuvants can trigger the symptoms of an allergic reaction even more commonly than the active substance itself. All ingredients contained in a vaccine are assumed to be disclosed in the ingredient list. However, small number of substances, including food proteins used as manufacturing aid during production process may not be considered medicine ingredients, for example egg used to culture vaccines [37]. Referring to the vaccines against SARS-Cov-2 that are currently approved all over the world including CoronaVac, Convidicea Ad5-nCoV, BBIBP-CorV-Sinopharm, Pfizer-BioNTech BNT162b2, Moderna mRNA-1273, ChAdOx1Oxford/AstraZeneca, Covaxin (BBV152), Sputnik V, EpiVacCorona, Janssen-Johnson & Johnson, it is important to underline that food allergens are not present in the excipient list [38,39,40]. In patients with food allergy, allergic reactions elicited by COVID-19 vaccines are not due to exposure to foods to which they are sensitized.

The second question is whether subjects with cow’s milk allergy or food allergy are at high risk for systemic reactions following COVID-19 vaccines. So far, limited data are available on this issue. Food allergies affect approximately 5% of adults and 8% of children and appear to have increased in prevalence [41,42]. The most common allergic food in children is cow’s milk. As a matter of fact, the incidence of cow’s milk allergy (CMA) is estimated to be around 2–3%, decreasing to less than 0.5% in adults [43]. A spontaneous tolerance toward cow’s milk is acquired by most allergic infants before the 3 years of age [44]. It has been suggested that the process of resolution of CMA can be favored by the introduction of baked milk into the diet of the child [45]. Regarding mechanisms of food allergy, IgE-mediated reactions are due to the degranulation mast cells and basophils triggered by crosslinking of IgE antibodies bound to the membrane receptors with food allergens. They are characterized by quick onset manifestations (urticaria, angioedema, rash, vomiting, diarrhea, rhinitis, conjunctivitis, laryngeal edema, wheezing, anaphylaxis) that develop often within 15 min up to 2 h following food intake. Delayed reactions can be mixed IgE/non IgE-mediated or non IgE-mediated. Mixed IgE/non IgE-mediated include asthma, atopic dermatitis, and eosinophilic gastrointestinal diseases. Non IgE-mediated reactions such as proctocolitis, enteritis, food protein enterocolitis, gastro-esophageal reflux, are believed to be due to stimulation of T cells leading to inflammation of the intestinal mucosa and increased intestinal permeability [46]. Food allergens are usually proteins. In cow’s milk, whey proteins (β-lactoglobulin and α-lactalbumin) and the caseins (αs1-, αs2-, β-, and κ-casein) are involved [47]. With pharmaceutical manufacturing of both drugs and vaccines, several food-derived substances may be blended with the active ingredient to improve stability, give protection and support, modify availability, and ameliorate acceptability. Small amounts of food allergens in medications can rarely elicit severe allergic reactions in patients allergic to the relevant food. [48]. Children with egg allergy are at risk of developing reactions to vaccine for measles-mumps-rubella-varicella, rabies, influenza, yellow fever that contain egg proteins. Children with milk allergy can develop anaphylaxis to cow’s milk proteins that contaminate lactose in 40 mg intravenous methylprednisolone sodium succinate and fluticasone/salmeterol or laninamivir dry-powder inhalers. Alpha-gal that is contained in cetuximab, antivenoms, prosthetic heart valves, recombinant human coagulation factor VII, heparin, colloids can elicit allergic reactions in patients with alpha-gal allergy. Patients with gelatin allergy can present allergic reactions after intake of pork and bovine gelatin added as a stabilizer in vaccines, erythropoietin product, gelatin based hemostatic products, colloids, and suppositories.

Reports showed a variable rate of food allergy among recipients who presented anaphylaxis after COVID-19 vaccine [13,26,27,28,29,31,33,35]. Anaphylactic reactions were associated with history of allergy to different foods including cow’s milk, nuts, egg, shellfish, and tropical fruits. Rojas-Pérez-Ezquerra P et al. [49] prospectively investigated 131 patients with previous severe allergic reactions to determine whether mRNA COVID-19 vaccines could be safely given. Patients had an history of anaphylaxis (121/131,92.4%) to drugs (66/121, 54.5%), foods (40/121, 33.1%), hymenoptera venom (4/121, 3.3%), latex (4/121, 3.3%), other allergens (10/121, 8.3%) or idiopathic (3/121, 2.5%). Nine (20%) patients had severe asthma, 7 patients (5.3%) chronic urticaria, and 1 patient (0.8%) mast cell activation syndrome. Skin prick tests with the mRNA vaccine and trometamol always resulted negative. Skin prick tests with PEG-3350 were positive in 2 women (1.6%). A woman had a history of anaphylaxis to drug and of idiopathic anaphylaxis. The second woman experienced anaphylaxis during vaginal delivery with epidural anesthesia. They did not receive the vaccine. Among patients who received mRNA COVID-19 vaccines, a woman with severe asthma developed rhinitis and skin rash. All other vaccinations were uneventfully. A limitation of the study was the low number of adults with food allergy that were enrolled. It is also of note that anaphylactic reactions have been described in subjects without any history of allergic disease as reported in the available studies, 19–57% cases of anaphylaxis after administration of Pfizer-BioNTech [26,35] and 10–22% after Moderna COVID 19 vaccine [25,35]. Correspondingly, due to insufficient data, at the beginning of the vaccination campaign, the UK regulator stated that prior anaphylaxis to a vaccine, medicine or food was a contraindication [27], but three weeks later, this recommendation was retired thanks to more reassuring data [28]. Neither US Food and Drug Administration (FDA) nor US Centers for Disease Control and Prevention (CDC) proposed the same contraindication, though they recommended to perform a prolonged observation following vaccination and to ensure the presence of trained staff and suitable facilities in all vaccination centers [29]. To date, allergy to food, insect venoms, drugs or inhalant allergens does not represent a contraindication for COVID-19 vaccines [40]. Patients suffering from allergic rhinitis or controlled asthma do not show any higher risk [40].

The third question is whether it is possible to reduce the risk of an anaphylactic reaction to vaccines. Anaphylaxis is a severe, life-threatening systemic allergic reaction that can also occur after vaccination, though rarely with an onset usually within minutes to hours. Nowadays, recipients who are predisposed to anaphylaxis elicited by a COVID-19 vaccine because of allergic reactions include those who are allergic to one of the COVID-19 vaccine components or who have developed an allergic reaction to a previous shot of a vaccine with similar vectors. However, CDC data showed that 84% (147/175) of recorded severe “allergic” reactions were not confirmed as anaphylaxis after case review [26]. Panic or anxiety reactions can mimic anaphylaxis, with symptoms such as flushing, tachycardia, shortness of breath or hypotension [50]. Therefore, an accurate screening for contraindications before administering COVID-19 vaccines is mandatory. The allergist should assess patients with unclear history of food allergy to ascertain by definitive means whether they are truly allergic [51]. It is worth of mention that severe allergic reactions are not always predictable, and they can occur in patients without previous episodes of anaphylaxis, history of allergic symptoms or hypersensitivity reactions to cow’s milk or other allergens. All individuals, especially with allergy to cow’s milk or other foods, should receive vaccines in a health-care infrastructure with available treatment for immediate allergic reactions and trained medical staff [40]. Personnel should be able to early recognize signs and symptoms of anaphylaxis and to promptly administer adrenaline intramuscular in the thigh that is the first therapeutic option. Another adrenaline dose should be administered after 5 min if required together with oxygen and intravenous fluid resuscitation. Emergency medical services and intensive care unit should be quickly available [40]. Second-line treatments include antihistamines for mucosal swelling, urticaria and oculorhinitis, and corticosteroids for urticaria, angioedema, asthma, and oculorhinitis. In vaccines, mRNA is wrapped in lipid nanoparticles that are attached to polyethylene glycol (PEG) molecules to augment stability and lifetime. PEG is widely used in cosmetics and pharmaceuticals and anti-PEG IgM and IgG antibodies were measured in 72% of people [52]. Anti-PEG IgE antibodies have been found in subjects allergic to drugs or mRNA vaccine containing PEG [33,53]. Even if, it is unclear whether anti-PEG IgE antibodies can trigger the allergic reaction to mRNA vaccines [54,55], so far, all treatments might prudently be PEG free. Understanding the rate of anti-PEG antibodies after first dose of vaccine can be helpful. The release of tryptase mainly from mast cell, occurs both in IgE-mediated and non IgE-mediated anaphylaxis. Increased serum tryptase levels, >1.2x baseline + 2 ng/L, help the diagnosis of anaphylactic reaction [56]. However, anaphylaxis can appear when levels remain normal [38]. Serum tryptase concentration should be measured 0.5–2 h up to 4 h after onset of the reaction, and at baseline, >24 h following resolution. In patients with history of severe allergic reactions, it is advised by national agency [29]. and scientific societies [40]. that the usual observation period of 15 min after vaccination should be lengthened to 30 min [55]. Unless it is required by patient’s history and needing, there is no clear evidence that it would be necessary to extend this period because of potential onset of delayed systemic symptoms, including wheezing or antibody-mediated responses such as type II and type III reactions due to IgM and IgG. Subjects with systemic allergic reaction to a vaccine should be referred to an allergist to investigate potential trigger. Local symptoms appeared in up to 80% of individuals after vaccination in the clinical trials. Recipients are not precluded from getting the second dose of COVID 19 vaccine even if large local reactions with pain, itching or swelling at the site of injection had appeared at the first dose. With reference to Moderna vaccine, delayed local hypersensitivity reactions [56] occurring after day 8 from vaccination, have been reported. Nevertheless, the completion of the whole cycle of vaccination must be encouraged.

## 4. Unmet Needs

Further investigations are warranted to determine a correct risk stratification for the pediatric population with a history of allergic reactions or anaphylaxis to foods. A study on the safety of COVID-19 vaccines in a large sample of subjects with allergy to cow’s milk or other foods is necessary to identify children who may be at risk for allergic reaction with vaccines. It would also be useful for assessing clinical or laboratory markers predicting adverse reactions and classifying children who need a longer observation period, an allergist’s consultancy or getting shot in a particular medical setting. Until these studies become available, it is impossible to exactly assess the impact of vaccines on people with food allergies. Patients with mastocytosis are potentially at higher risk of food allergy as well as increased release of mast cell mediators by non IgE-mediated mechanisms. So far, mRNA COVID-19 vaccines have been shown to be well tolerated in patients with mastocytosis and anaphylaxis [57]. Larger clinical trials are warranted to establish whether mRNA COVID-19 vaccines are safe to people with mastocytosis and cow’s milk allergy. In patients who experienced systemic allergic reactions with the first dose of COVID-19 vaccination, the second dose of the vaccine should not be given [40,55]. A path for allergy work-up of COVID-19 vaccination in allergic patients has been proposed [40]. It needs to be clarified the accuracy of allergy tests with vaccine and its components to exactly understand the triggers of allergic reactions to COVID-19 vaccines in patients with food allergy. Along this line, it should be ascertained by definitive means that traces of food proteins have not contaminated vaccines during production process [37]. It should be also investigated whether they may safely get the second shot with a vaccine with different excipients and active ingredient. Finally, the potential role of desensitization protocols can be assessed.

## 5. Conclusions

**Figure 1:**
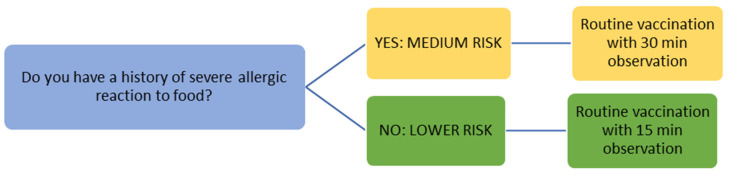
Vaccination in children with allergy to cow’s milk or other foods.

The COVID-19 vaccination campaign is one of the most important challenges of our times. For this reason, vaccination must proceed as quickly and safely as possible, for population suffering from allergic diseases too. Available data are limited both for the complete understanding of risk factors and for the pathogenetic mechanisms of allergic reaction to vaccines. It is therefore necessary to continuously monitor the safety of COVID-19 vaccines. A precise and detailed report of any possible allergic adverse reaction should be always obtained to make vaccination safer and more accessible to each category of patients. Current evidence indicates that patients with a history allergy to cow’s milk or foods even if severe, should get COVID-19 vaccine in a setting with availability of emergency treatment and under medical supervision. It is suggested [29,40,55] that recipient can be discharged after an observation period of 30 min if no reaction developed (Figure 1). Up to now, data are referred to adults and adolescents, but clinical trials are being carried out to evaluate the safety and efficacy in younger children.

## Figures and Tables

**Table 1:** Type of potential triggers of hypersensitivity reactions that are contained in vaccines to infectious diseases [20,21]. * PEG in this formulation is a carrier and not an excipient. It is conjugated to one of four lipids that make up the PEGylated lipid nanoparticles. Vaccines based on mRNA use lipid nanoparticles to facilitate the transport of mRNA into cells.

Type	Excipient	Vaccine Type	Hypersensitivity Reaction
Adjuvant	ASO-3	AS0-3 adjuvanted A/H1N1 pandemic influenza vaccine	Immediate
	Aluminium	DTaP/Tdap/DT/MEN B/HepB/HepA/HPV/DTaP + IPV/DTaP + IP V + HepB/Hib + HepB/DTaP + IPV + Hib/PCV 13/SARS-CoV-2	Delayed (small granulomas, nodules)
Preservative	Thimerosal	DT/Td/influenza/Japanese encephalitis/Menigococcus	Delayed, systemic allergic reaction (rare)
Antimicrobial	Neomycin	Influenza/HepA/IPV/DTaP + IPV/MMR/D TaP + HepB + IPV/DTaP + IPV + Hib/MMRV/DTaP + IPV/Rabies/HepA/Varicella/Influenza/HepA + HepB	Immediate and delayed
	Gentamicin Sulfate	Influenza	
	PolymixinB	Influenza/IPV/DTaP + IPV/DTaP + HepB + IPV	
Surfactant	Polysorbate 20	Influenza/HepA/HepA + HepB	Immediate
	Polysorbate 80	HPV/influenza/HepB/DTaP/Japanese Encephalitis/DTaP + IPV/DTaP + HepB + IPV/DTaP + IPV + Hib/PCV13/Rotavirus/MEN B/COVID-19/Zooster/SARS-CoV-2 (AstraZeneca, J&J)	
Carrier, Polyethylene Glycol *		SARS-CoV-2 (Moderna, Pfizer)	
Residual Medium	Egg (ovoalbumin, egg protein)	Influenza, MMR/MMRV, YF, TBE, Rabies.	Minor/local, anaphylaxis (rare)
Manufacturing residue/stabilizer	Gelatin	YF/MMR/MMRV/Varicella/influenza/Zooster/Japanese encephalitis/TBE	Immediate and delayed
Pharmaceutical closure	Latex	Tdap/Menigococcal/Hip + HepB/HepB/Influenza/HepA/HepB + HepA/DTaP/DTaP + IP V/DTaP + HepB + IPV/Rotavirus/Td	Immediate
Medium nutrient	Yeast (Saccharomyces cerevisiae)	Hib + HepB/HepB/HPV/Meningococcal/DTaP + HepB + IPV/PCV13/HepB/HepA + HepB/Typhoid	Immediate
	Cow’s milk	DTaP/Td/Tdap/OPV/Typhoid fever (oral)/MMR	Immediate

Abbreviations: AS0: squalen-based adiuvant; DT: diphtheria, tetanus; DTaP: diphtheria, tetanus and acellular pertussis; HepA: Hepatitis A; HepB: Hepatitis B; Hib: Haemophilus influenzae type b; HPV: Human papillomavirus; IPV: inactivated polio vaccine; MMR: Measles, Mumps, Rubella; MMRV: Measles, Mumps, Rubella, Varicella; OPV: oral polio vaccine; PCV13: Pneumococcal 13-valent; SARS-CoV-2: severe acute respiratory syndrome coronavirus-2; TBE: thick born encephalitis; Td:Tetanus and Diphtheria Toxoids adsorbed; Tdap: tetanus, reduced diphtheria and acellular pertussis; YF: yellow fever.
